# Anti-trypanosomal, anti-inflammatory, and neuroprotective effects of *Cichorium intybus* sesquiterpene lactones in experimental *Trypanosoma evansi* infection

**DOI:** 10.1038/s41598-026-47119-z

**Published:** 2026-04-27

**Authors:** Marian G. Sawerus, Hamdy H. Kamel, Walaa M. S. Ahmed, Sobhy Abdel-Shafy, Dalia El Amir, Emad A. Mahdi, Marwa A. Ibrahim, Olfat Shehata

**Affiliations:** 1 Department of Clinical Pathology, Faculty of Veterinary Medicine, Beni-Suef University, Beni-Suef, 62511 Egypt; 2 Department of Parasitology and Animal Diseases, Veterinary Research Institute, National Research Centre, Dokki, 12622 Giza Egypt; 3 Department of Pharmacognosy, Faculty of Pharmacy, Beni-Suef University, Beni-Suef, 62514 Egypt; 4 Department of Pathology, Faculty of Veterinary Medicine, Beni-Suef University, Beni-Suef, 62511 Egypt; 5 Department of Biochemistry and Molecular Biology, Faculty of Veterinary Medicine, Cairo University, Giza, 12211 Egypt

**Keywords:** *Trypanosoma evansi*, Chicory, Sesquiterpene lactones, Rat, Oxidative stress, Diseases, Drug discovery, Immunology, Microbiology

## Abstract

*Trypanosoma evansi* is an important protozoan pathogen affecting various animal species particularly camels and horses, leading to significant economic losses. The ineffective control of *T. evansi* is attributed to the absence of vaccines and emergence of resistance towards existing chemotherapeutic agents. This study evaluated the efficacy of a fraction enriched with sesquiterpene lactones derived from *Cichorium intybus* on parasitemia levels and related pathological changes in *Trypanosoma evansi*-infected rats. Eighty healthy male albino rats were allocated into four groups of equal size; Group I served as the control negative (uninfected). Group II received an intraperitoneal infection with *Trypanosoma evansi* (1 × 10⁴ trypanosomes/rat). Group III was also infected and received intramuscular treatment with diminazene aceturate (7 mg/kg body weight). Group IV administered a daily oral dose of *C. intybus* fraction (200 mg/kg body weight) and similarly infected. Infection by *T. evansi* induced anemia (normocytic normochromic), leucocytosis, alterations in lipid profile, and hypoglycemia. Furthermore, the infection resulted in the upregulation of interleukin-1β (IL-1β) and interleukin-6 (IL-6), alongside the downregulation of transforming growth factor-beta (TGF-β) and interleukin-10 (IL-10) gene expression levels. The pathological lesions in the splenic and brain tissues, coupled with a significant increase in brain acetylcholinesterase (AChE) activity and oxidative stress, were also observed in the infected groups. *Cichorium intybus* fraction administration significantly reduced the parasitemia level and alleviated the pathological changes caused by *T. evansi* compared to the untreated infected group. The sesquiterpene lactones- enriched fraction from *C. intybus* demonstrates a notable anti-trypanosomal efficacy and may represent a promising source for the development of novel trypanocidal compounds.

**Supplementary Information:**

The online version contains supplementary material available at 10.1038/s41598-026-47119-z.

## Introduction

Trypanosomiasis is induced by *Trypanosoma* species and the predominant causative agent in camels is *Trypanosoma evansi* (*T. evansi)*, a salivarian trypanosome that is spread by mechanical transmission by biting flies, predominantly Stomoxys and Tabanids. The disease induced by this pathogen is known as Surra^1,2^. This protozoan exhibits an extensive host range, that includes camels, equines, dogs, buffalo, cattle, goats, and sheep. In particular, a severe form of infection is frequently observed in equines, camels, buffalo, and cattle^3,4^. *T. evansi* represents a significant protozoan disease impacting camels, exerting a substantial economic impact within endemic regions, primarily through diminished production of meat and milk, as well as lowered work efficiency in camel husbandry sectors. Global epidemiological data indicates a widespread distribution of this protozoan, with a marked prevalence observed in numerous countries in the Middle East^1^.

The disease typically manifests with clinical signs such as anemia, pyrexia, linked to parasitemia, loss of body weight, and intermittent fever due to repeated episodes of the parasitemia during the course of the disease. Observations often include hemorrhages within the mucous membranes, rough coats, urticarial plaques, and edema. Additionally, vaccination failures are frequently indicative of immunosuppression^1,3^.

The extensive antigenic diversity exhibited by trypanosomes enables all *Trypanosoma*species to efficiently evade host immune responses, substantially reducing the likelihood of developing an effective universal vaccine in the future^5^. Thus, chemotherapy is the main method to control trypanosomiasis. Most of the chemotherapy employed in the treatment of *T. evansi* demonstrates limited efficacy, frequently leading to the recurrence of parasitemia and related clinical signs. Five drugs, namely melarsomine, quinapyramine, isometamidium chloride, suramin, and diminazene aceturate, are utilized in the treatment of Surra today. These drugs are costly and not universally available. The emergence of drug resistance is considered a major threat to the efficacy of these medications and emphasizes the critical need for novel therapeutic agents^6–8^.

Plant products are thought to be a rich source of new anti-parasitic components and have been applied in the treatment of a variety of diseases since ancient times^9^. *Cichorium intybus* (*C. intybus*), commonly referred to as chicory, is a Mediterranean plant of the Asteraceae family, widely cultivated in Europe, Asia, and Egypt. This species is recognized for its high content of terpenes, particularly those belonging to the sesquiterpene lactones (STLs) group, and it also comprises a variety of compounds with medicinal significance, such as coumarins, caffeic acid derivatives, fatty acids, alkaloids, amino acids, flavonoids, vitamins, and minerals^10^.

*C. intybus* exhibits diverse medicinal properties, including anti-inflammatory, anticancer, antioxidant, antidiabetic, antimicrobial, antihyperlipidemic, immunomodulatory, antinociceptive, hepatoprotective, neuroprotective, and cardioprotective effects^11^. Consequently, it is extensively employed in the treatment of various ailments such as fever, jaundice, gout, hepatic disorders, splenic enlargement, and rheumatoid arthritis^12–14^. The STLs and their derivatives thought to be responsible for these activities^15,16^. In addition, the *in vitro* anti-protozoal activity of *C. intybus* root and leaf extracts against *Cryptosporidium parvum* had been investigated by Woolsey et al.^17^ and the extracts showed a dose-dependent inhibition in the growth.

According to recent reports, *C. intybus* is a potentially rich source of novel antiparasitic compounds and has been increasingly investigated for anthelmintic activities^18,19^. However, its efficacy on protozoan parasites is poorly investigated, and limited studies have demonstrated its trypanocidal potential^17^. Pena-Espinoza et al.^20^ investigated the trypanocidal efficacy of *C. intybus* against *Trypanosoma cruzi (T. cruzi) in vitro* and predicted trypanocidal constituents via bioactivity-based molecular networking and metabolomic analyses. They found that eleven compounds, including the sesquiterpene lactone lactucin, fatty acid derivatives, and flavonoids, displayed potent activity against *T. cruzi*.

Hence, the aim of this study was to assess the trypanocidal efficacy of *C. intybus* sesquiterpene lactones-enriched fraction as well as the antioxidant potential, along with the resultant clinical outcomes in rats experimentally infected with *T. evansi*. According to our knowledge, this is the first* in vivo* study that investigates the efficacy of *C. intybus* sesquiterpene lactones-enriched fraction against *T. evansi*.

## Materials and methods

### Plant material and chemicals

The leaves of *C. intybus* were purchased from the Harraz^®^ market (Cairo, Egypt). The plant material was identified by Dr. Ahmed M. Ayyat (Department of Medicinal and Aromatic Plant Faculty of Agriculture, Beni-Suef University 62521, Egypt; Research Institute of Medicinal and Aromatic Plants (RIMAP), Beni-Suef University 62521, Egypt).

As part of our earlier study^21^, we extracted, fractionated, and analyzed the *C. intybus* sesquiterpene lactones-enriched fraction using liquid chromatography–mass spectrometry (LC–MS). The previously prepared extract was then used in the present study. The sample was ground into a fine powder (4.5 kg). Ethanol: water (80:20, v/v) was used for the extraction by ultra-sonication for 15 min at room temperature, followed by a 10-min centrifugation at 11,000xg. The collected supernatant was filtered, and the solvent was evaporated till dryness. The residue (420 gm) was extracted with dichloromethane (DCM) till exhaustion, followed by solvent evaporation till dryness yielding 70 gm (15 gm/kg dry wt.)^21^. Before LC-MS analysis, 1 g of the extracted fraction was dissolved in methyl alcohol and filtered by a 0.22 μm membrane filter. Rats were given the fraction orally after dissolving it in 2% Tween 80.

Chemicals for assessing oxidative stress biomarkers and AChE activity were bought from Carl Roth-Germany and Loba-chemie Company, Mumbai, India. BATRYNIL^®^ (Arabcomed, Arab Company for Medical Products, Egypt), containing diminazene aceturate and phenazone), was used in this study.

### *Trypanosoma evansi*

*Trypanosoma evansi* strain was originally isolated from infected camels and cryopreserved in liquid nitrogen using a medium composed of 1× PBS, 1% glucose, and 1% Dimethyl sulfoxide (DMSO). A relatively low concentration of DMSO (1%) was used in the cryopreservation medium because the parasite isolate was stored for maintenance prior to experimental infection rather than for long-term archival preservation. Using this protocol, the *T. evansi* isolate maintained its viability and infectivity after storage in liquid nitrogen for up to one year. Based on the preliminary study, the used isolate is able to induce an acute infection with 13–15 days-survival period following infection in rats, if not treated. Initially, cryopreserved blood was intraperitoneally injected into two rats to produce a large number of trypanosomes for the experimental groups’ subsequent infection in this study.

### Animals and experimental design

Eighty healthy male rats weighing between 170 and 200 g were purchased from the Nile Company (El Amyria, Cairo, Egypt) and acclimated for 10 days under controlled environmental conditions, which included a regulated light cycle (12 h day/night cycle), temperature (25 ± 2 °C), and humidity (60 to 70%). The rats were provided with unrestricted access to water and food to assure highest animal welfare conditions.

The experiment was approved by the Research Ethical Committee of Beni-Suef University-Faculty of Veterinary Medicine (approval number 022–296). All methods we carried out in accordance with relevant guidelines and regulations. The study followed the recommendations of the ARRIVE guidelines (Animal Research: Reporting of In Vivo Experiments). The experimental period was one month and the treatment regimens for chicory fraction, commenced 2 weeks before infection and another 2 weeks post-infection. Rats were randomly allocated equally into 4 groups (*n* = 20/group). Group I was the control negative, which was untreated and uninfected. Group II was control positive and received an intraperitoneal infection with *T. evansi* (1 × 10^4^ trypanosomes/rat)^22^ on the 15^th^ day from the beginning of the experiment. Group III was infected as described and treated intramuscularly with 7 mg/kg body weight of diminazene aceturate (standard chemical drug) as a single dose^23^ on the 8^th^ day post-infection (when all the rats, in the infected groups, were parasitemic). The chicory fraction group (IV) was orally treated with 200 mg/kg body weight of sesquiterpene lactones-enriched fraction from *C. intybus* and infected as described (Supplementary Fig. 1). The chicory fraction was given in a daily 1 ml volume per rat for 4 weeks (2 weeks before infection and 2 weeks after infection). The pre-infection administration of the chicory fraction was designed to evaluate both the prophylactic and early therapeutic potential of the fraction components. The other groups (I, II, III) in the experiment were orally treated with saline (1 ml/rat/day) during the experimental period.

The chicory fraction dosage was determined based on the acute toxicity study^21^, following the Organization for Economic Co-operation and Development (OECD) guideline number 423. A dose of 200 mg/kg body weight of chicory fraction (corresponding to one-tenth of 2000 mg/kg body weight) was chosen as the highest safe dosage that can be daily given for a long time.

### Estimation of parasitemia

A daily hematological assessment was conducted post-infection for all infected rats to detect the appearance of trypanosomes via wet preparation (a glass slide with a drop of blood from a tail vein was covered with a slide cover and microscopically examined). As *T. evansi* is a monomorphic trypomastigote and has lost the ability to complete its life cycle in insect vectors, its development is locked in the bloodstream form. After the parasite detection in the wet preparation (on the 7^th^ and 8^th^ DPI), the parasitemia degree was estimated using hemocytometer on the 10^th^, 12^th^, and 14^th^ DPI in all infected groups (*n* = 5/time point) following the previous methodology outlined by Dyary et al.^24^.

$$\:\mathrm{T}\mathrm{r}\mathrm{y}\mathrm{p}\mathrm{a}\mathrm{n}\mathrm{o}\mathrm{s}\mathrm{o}\mathrm{m}\mathrm{e}\mathrm{s}\\\mathrm{c}\mathrm{o}\mathrm{u}\mathrm{n}\mathrm{t}\:(\mathrm{n}\mathrm{u}\mathrm{m}\mathrm{b}\mathrm{e}\mathrm{r}/\mathrm{m}\mathrm{l})=\frac{N}{4}\times\:\mathrm{D}\mathrm{i}\mathrm{l}\mathrm{u}\mathrm{t}\mathrm{i}\mathrm{o}\mathrm{n}\:\mathrm{f}\mathrm{a}\mathrm{c}\mathrm{t}\mathrm{o}\mathrm{r}\times\:{10}^{4}$$

* N is the total number of trypanosomes in the four large squares.

All parasite counts were performed by the same trained person under standardized conditions to minimize variability.

### Sampling

Five rats were selected randomly (ensuring unbiased representation) from each group and underwent deep anesthesia by isoflurane on zero day (prior to the challenge) and on the tenth- and fourteenth-day post infection (DPI) in order to collect blood samples. Following the blood collection, the animals were euthanized humanely via isoflurane overdose.

A portion of the blood specimens was collected on EDTA, as an anticoagulant, for analyzing gene expression of IL-6, IL-1β, TGF-β, and IL-10 and relevant hematological parameters. For conducting biochemical analysis, the remaining portion of blood specimens was collected in tubes without an anticoagulant for the separation of the serum. The serum specimens were kept at −20℃ till analysis.

Following the animals’ euthanasia, on the 14^th^ DPI, spleen and brain were collected post-mortem. The brain tissues underwent a saline wash and allocated into 2 parts; the first one was homogenized to estimate oxidant/antioxidant parameters and the activity of AChE. The second part of the brain and the whole spleen were kept in formalin solution (10%) for histopathological analysis^25^ (Supplementary Fig. 1).

### Hematology

On zero day as well as the 10^th^, and 14^th^ DPI, the total erythrocyte and leucocyte counting was conducted via haemocytometer method^26^. The blood Hb and packed cell volume (PCV) were estimated as described by Drabkin and Austin^27^ and Thrall et al.^28^, respectively. Blood indices involving mean corpuscular hemoglobin (MCH), mean corpuscular volume (MCV), as well as mean corpuscular hemoglobin concentration (MCHC), were calculated following Dacie and Lewis^29^. The absolute differential leucocytic count was calculated and conducted in accordance with Jain^30^.

### Biochemistry

The concentrations of triglycerides, total cholesterol (TC), high-density lipoprotein cholesterol (HDL-C), and serum glucose were estimated using commercial kits, in accordance with the manufacturer’s protocol on zero day, 10^th^, and 14^th^ DPI. The levels of very low-density lipoprotein cholesterol (VLDL-C) and low-density lipoprotein cholesterol (LDL-C) were calculated by Friedewald’s formula (VLDL-C = Triglycerides/5, and LDL-C = TC - (VLDL-C + HDL-C)).

### Brain oxidant/antioxidant parameters and the activity of AChE

In phosphate buffer saline (pH 7.4), the brains were homogenized at a 10% (w/v) concentration, and centrifuged at 1000 xg at 4 °C for 15 min. The resulting supernatant was isolated and utilized for the oxidant/antioxidant parameters and AChE activity measurements.

The malondialdehyde (MDA) content was estimated calorimetrically, in brain homogenate, following the method of Albro et al.^31^. The methodology depends on the thiobarbituric acid (TBA) reagent reacting with MDA in an acidic medium, forming a red color. A 1.25 ml of 10% trichloroacetic acid (TCA) was mixed with 0.25 ml of brain homogenate in a glass tube and incubated in boiling water for 20 min. After incubation, the tubes were cooled in tap water and 0.5 ml D.W was added to each tube, then the tubes were centrifuged at 1800 xg for 10 min. 1 ml of the supernatant was mixed with 0.5 ml of 0.67% TBA, then the tubes were incubated in boiling water for 20 min. The tubes were cooled in tap water and the optical density was determined at 532 nm against a TBA blank. The content was expressed as nM/100 mg tissue.

By using DTNB (5,5′-dithiobis-2-nitrobenzoic acid), the reduced glutathione (GSH) content and glutathione peroxidase (GPx) activity were determined as reported by Sedlak and Lindsay^32^ and Rotruck et al.^33^, respectively. For estimation of GSH content, the technique involved in protein precipitation by TCA. 250 µl of 10% TCA was mixed with 250 µl of tissue homogenate in a glass tube for 10 min. The tubes were centrifuged at 1800 xg for 10 min. The supernatant (250 µl) was mixed with 125 µl of Ellman’s reagent (19.8 mg DTNB in 100 mL 0.1% sodium nitrate) and 750 µl of phosphate buffer and the optical density was read immediately at 412 nm against the blank. The content was expressed as nM/100 mg tissue. For GPx activity, the reaction mixture contained 100 µL 0.4 M phosphate buffer (pH 7.0), 50 µL 10 mM sodium azide, 100 µL tissue homogenate, 100 µL 2 mM reduced glutathione and 50 µL 0.2 mM H_2_O_2_. The contents were incubated for 10 min at 37 °C, 200 µL 10% TCA was added to stop the reaction and centrifuged at 1800 xg for 10 min. The supernatant was assayed for glutathione content using Ellman’s reagent. The activity was expressed as nM of GSH consumed/min/100 mg tissue.

The activity of AChE was measured using DTNB as the procedure of Ellman et al.^34^ and the modification mentioned by Gorun et al.^35^. Brain homogenates (0.01 ml) were incubated with 0.05 ml of acetylthiocholine solution for 30 min and the enzyme activity was stopped by the addition of 1.8 ml of DTNB reagent in ethanol (12.4 mg DTNB dissolved in 120 ml of 96% ethanol, 80 ml of D.W and 50 ml of 0.1 M phosphate buffer, pH 7.6). The absorbance was measured immediately at 412 nm. The principle is based on measurement of the thiocholine production rate as a result of acetylthiocholine hydrolysis and the result was expressed as nM thiocholine produced/min/100 mg tissue.

### Quantitative real-time PCR of inflammatory cytokines

The Qiagen RNeasy kit was used to extract total RNA from blood samples following the manufacturer’s instructions. The RevertAid First Strand cDNA Synthesis Kit was used to convert the isolated RNA to cDNA, and a real-time PCR system (Applied Biosystems, USA) was used to quantify the levels of IL-6, IL-1β, TGF-β, and IL-10 gene expression using SsoAdvancedTM Universal SYBR^®^ Green Supermix following the manufacturer’s guidelines. For the normalization of gene expression data by the f0 method, the beta-actin gene (Actb) was utilized as a housekeeping gene^36^. Beta-actin was used as the internal control due to its stable expression under the experimental conditions. The primers used in the present study are presented in Supplementary Table 1.

### Statistical analysis

The data analysis was conducted utilizing IBM SPSS Version 26. To elucidate significant intergroup differences at the same time point, a one-way analysis of variance complemented by the Tukey post-hoc test was employed. Data are presented as means accompanied by standard error (SE), with statistical significance established at *P* < 0.05.

## Results

### LC-MS analysis of *Cichorium intybus* sesquiterpene lactones-enriched fraction

The LC-MS analysis of the *C. intybus* sesquiterpene lactones-enriched fraction provides a comprehensive analysis of its chemical composition. Supplementary Table 2 lists the specific components identified within this fraction, providing an overview of its phytochemical profile.

### Parasitemia degree

**Fig. 1:**
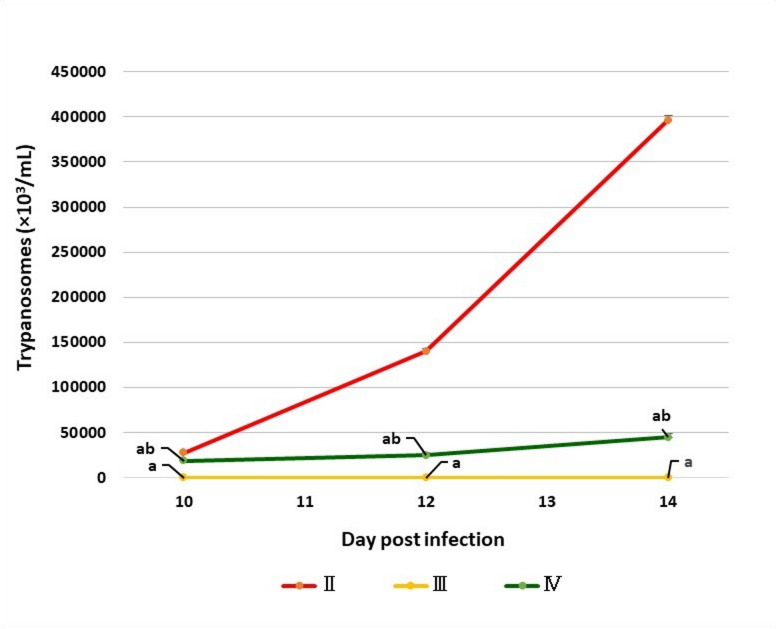
The degree of parasitemia in infected groups. The values are presented as means ± SE (*n* = 5) with different letters: (**a**) significant difference from group II; (**b**) significant difference from group III; significant difference at *P < 0.05*. II: control positive (infected) group; III: infected and treated with diminazene aceturate group; IV: treated with chicory fraction and infected group. DPI day post-infection.

In all groups subjected to infection, the prepatent period was observed between the 7^th^ and 8^th^ DPI. Rats in Group II (infected and untreated controls) showed a progressive increase in parasitemia whereas, rats in Group III did not show parasites in their blood smears after being treated with diminazene aceturate up to the 14^th^ DPI. Group IV showed a statistically significant decrease in parasitemia levels compared to Group II after receiving chicory fraction orally (Fig. 1). All rats in Group IV survived until the experimental endpoint at day 14 post-infection.

### Hematology

The erythrogram and leucogram on day 0 demonstrated no statistically significant differences amongst all experimental groups.

**Fig. 2:**
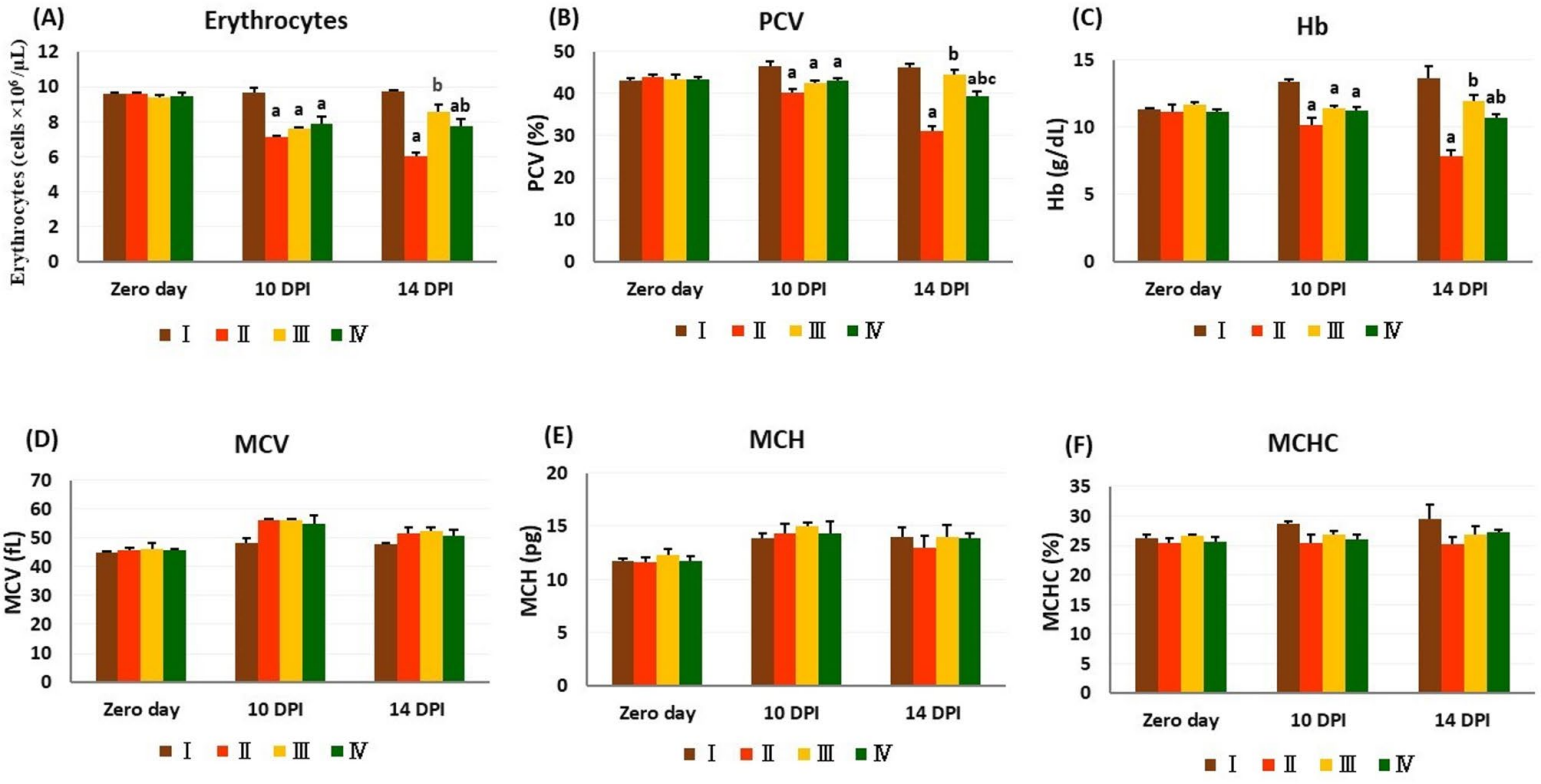
Erythrogram in experimental rats in different experimental groups. The values are presented as means ± SE (*n* = 5) with different letters: (**a**) significant difference from group I; (**b**) significant difference from group II; (**c**) significant difference from group III; significant difference at *P < 0.05*. I: control negative group; II: control positive (infected) group; III: infected and treated with diminazene aceturate group; IV: treated with chicory fraction and infected group. PCV packed cell volume, Hb hemoglobin, MCV mean corpuscular volume, MCH mean corpuscular hemoglobin, MCHC mean corpuscular hemoglobin concentration. DPI day post-infection.

On the 10^th^ DPI, there was a marked decrease in the erythrocyte, PCV, and Hb values, with normal blood indices, exhibiting normocytic normochromic anemia in *T. evansi*-infected groups in comparison to group I. On the 14^th^ DPI, the blood indices showed no statistically significant differences amongst the experimental groups. Specifically, in contrast to group I, a significant decrease in erythrocyte count, PCV, and Hb concentration was observed in groups II and IV. Conversely, these parameters displayed marked elevation in the treated groups (III and IV) compared to group II, with a more pronounced elevation in PCV values in group III than in group IV (Fig. 2).

On the 10^th^ DPI, all infected groups showed statistically significant leucocytosis, lymphocytosis, and monocytosis relative to group I. Additionally, group II displayed neutrophilia as opposed to group I. However, both treatment groups (III and IV) showed a significant decrease in total leucocytic count, neutrophils, lymphocytes, and monocytes in comparison to group II. On the 14^th^ DPI, groups II and IV experienced notable leucocytosis, coupled with lymphocytosis and monocytosis, relative to group I. Neutrophilia was observed only in group II compared to group I. In comparison to group II, the infection-induced leucocytosis, lymphocytosis, neutrophilia, and monocytosis were significantly reduced in groups III and IV, with a more noticeable reduction observed in group III than in group IV (Table 1).

**Table 1:** Leucogram in different experimental groups.

Parameters	Groups	WBCs (cells ×10^3^/µL)	Lymphocytes (cells ×10^3^/µL)	Neutrophils (cells ×10^3^/µL)	Eosinophils (cells ×10^3^/µL)	Monocytes (cells ×10^3^/µL)
Zero day	I	10.08 ± 0.20	8.18 ± 0.27	1.17 ± 0.04	0.22 ± 0.11	0.51 ± 0.01
	II	10.05 ± 0.17	8.10 ± 0.14	1.19 ± 0.04	0.30 ± 0.01	0.46 ± 0.08
	III	10.23 ± 0.09	8.10 ± 0.07	1.27 ± 0.04	0.35 ± 0.09	0.52 ± 0.01
	IV	10.27 ± 0.21	7.77 ± 0.29	1.61 ± 0.36	0.48 ± 0.03	0.41 ± 0.05
10^th^ DPI	I	8.83 ± 0.36	7.01 ± 0.31	1.18 ± 0.06	0.34 ± 0.03	0.31 ± 0.03
	II	16.84 ± 0.23 ^a^	12.44 ± 0.43 ^a^	2.60 ± 0.39 ^a^	0.51 ± 0.18	1.29 ± 0.07 ^a^
	III	11.57 ± 0.33 ^ab^	8.70 ± 0.28 ^ab^	1.65 ± 0.04 ^b^	0.45 ± 0.03	0.77 ± 0.04 ^ab^
	IV	12.60 ± 0.38^ab^	9.91 ± 0.26 ^ab^	1.56 ± 0.09 ^b^	0.34 ± 0.04	0.80 ± 0.05 ^ab^
14^th^ DPI	I	9.22 ± 0.39	7.41 ± 0.30	1.09 ± 0.01	0.25 ± 0.03	0.47 ± 0.07
	II	18.22 ± 0.14 ^a^	14.27 ± 0.23 ^a^	2.27 ± 0.09 ^a^	0.23 ± 0.05	1.45 ± 0.10 ^a^
	III	9.95 ± 0.56 ^b^	7.84 ± 0.42 ^b^	1.36 ± 0.10 ^b^	0.28 ± 0.01	0.47 ± 0.06 ^b^
	IV	12.85 ± 0.12 ^abc^	10.32 ± 0.03 ^abc^	1.33 ± 0.16 ^b^	0.34 ± 0.05	0.86 ± 0.04 ^abc^

The values are presented as means ± SE (*n* = 5) with different letters: (a) significant difference from group I; (b) significant difference from group II; (c) significant difference from group III; significant difference at *P < 0.05*. I: control negative group; II: control positive (infected) group; III: infected and treated with diminazene aceturate group; IV: treated with chicory fraction and infected group. WBCs white blood cells, DPI day post-infection.

### Biochemistry

On zero day, there were no significant differences in the biochemical parameter values amongst the experimental groups compared with uninfected controls. Conversely, on the 10^th^ DPI, all infected groups showed significant hypoglycemia along with elevated triglycerides and VLDL-C. These variations were maintained only in groups II and IV on the 14^th^ DPI in comparison to group I. At both time points, the treated groups (III and IV) showed a significant decrease in both levels of triglycerides and VLDL-C, coupled with the significant increase in blood glucose levels relative to group II. In comparison to group IV, group III displayed a considerable decrease in triglycerides and VLDL-C levels on the 10^th^ and 14^th^ DPI and a notable increase in glucose levels only on the 14^th^ DPI.

**Fig. 3:**
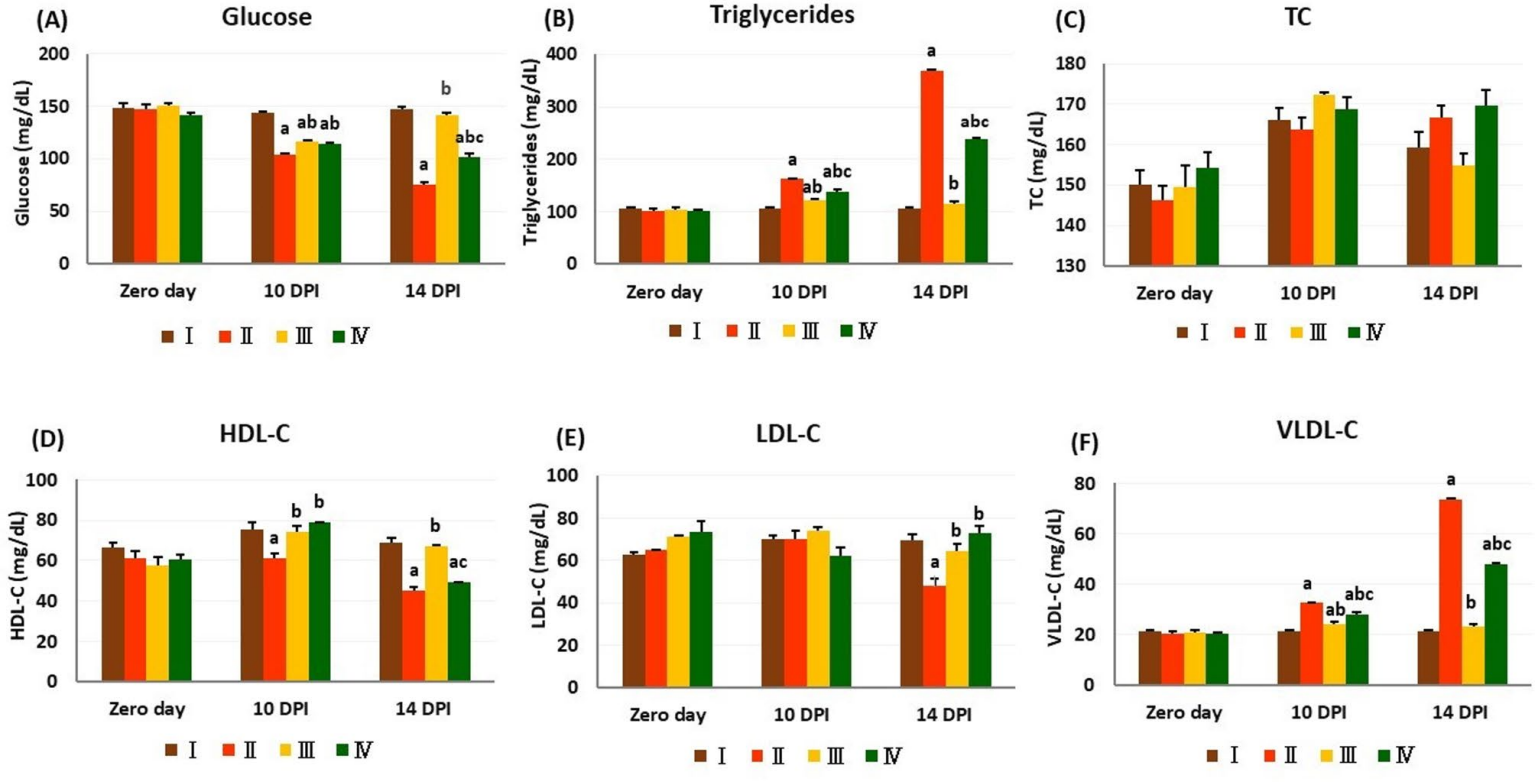
Biochemical parameters in different experimental groups. The values are presented as means ± SE (*n* = 5) with different letters: (**a**) significant difference from group I; (**b**) significant difference from group II; (**c**) significant difference from group III; significant difference at *P < 0.05*. I: control negative group; II: control positive (infected) group; III: infected and treated with diminazene aceturate group; IV: treated with chicory fraction and infected group. TC total cholesterol, HDL-C high-density lipoprotein cholesterol, LDL-C low-density lipoprotein cholesterol, VLDL-C very low-density lipoprotein cholesterol, DPI day post-infection.

The HDL-C concentration was considerably reduced only in group II on the 10^th^ DPI. However, on the 14^th^ DPI, its concentration was significantly reduced in the groups II and IV compared to the other groups. Furthermore, on the 14^th^ DPI, the LDL-C level significantly reduced in group II, compared to the remaining experimental groups (Fig. 3).

### Brain oxidant/antioxidant parameters and the activity of AChE on the 14th DPI

All infected animals showed a statistically significant decline in the reduced GSH concentration and the activity of GPx; however, only groups II and III demonstrated significantly high levels of MDA content and AChE activity when compared to group I. The reduced GSH concentration, besides the activities of GPx and AChE, was significantly improved in both III and IV groups when compared to group II. Group IV showed a greater improvement in the activity of GPx and AChE than did group III (Table 2).

**Table 2:** Brain oxidant/antioxidant parameters and the activity of AChE in different groups on the 14^th^DPI.

Parameters Groups	MDA nM/100 mg tissue	GSH nM/100 mg tissue	GPx nM GSH/min/100 mg tissue	AChE nM thiocholine/min/100 mg tissue
I	10.69 ± 0.70	41.92 ± 1.23	71.32 ± 1.54	22.32 ± 0.14
II	14.59 ± 0.51 ^a^	29.56 ± 0.62 ^a^	40.93 ± 0.82 ^a^	29.97 ± 0.23 ^a^
III	13.76 ± 0.76 ^a^	32.20 ± 0.29 ^ab^	55.21 ± 0.65 ^ab^	27.81 ± 0.13 ^ab^
IV	12.41 ± 0.27	33.09 ± 0.66 ^ab^	64.57 ± 1.09 ^abc^	22.71 ± 0.33 ^bc^

The values are presented as means ± SE (*n* = 5) with different letters: (a) significant difference from group I; (b) significant difference from group II; (c) significant difference from group III; significant difference at *P < 0.05*. I: control negative group; II: control positive (infected) group; III: infected and treated with diminazene aceturate group; IV: treated with chicory fraction and infected group. DPI: day post-infection.

MDA malondialdehyde, GSH reduced glutathione, GPx glutathione peroxidase, AChE acetylcholinesterase.

### Inflammatory cytokines gene expression levels

The chicory fraction administration led to a significant decline in the level of IL-6 mRNA on zero day, relative to other experimental groups. The infection with *T. evansi* significantly increased IL-1β and IL-6 mRNA expression levels while reducing TGF-β and IL-10 mRNA expression levels across all infected groups, compared to group I on the 10^th^ and 14^th^ DPI.

**Fig. 4:**
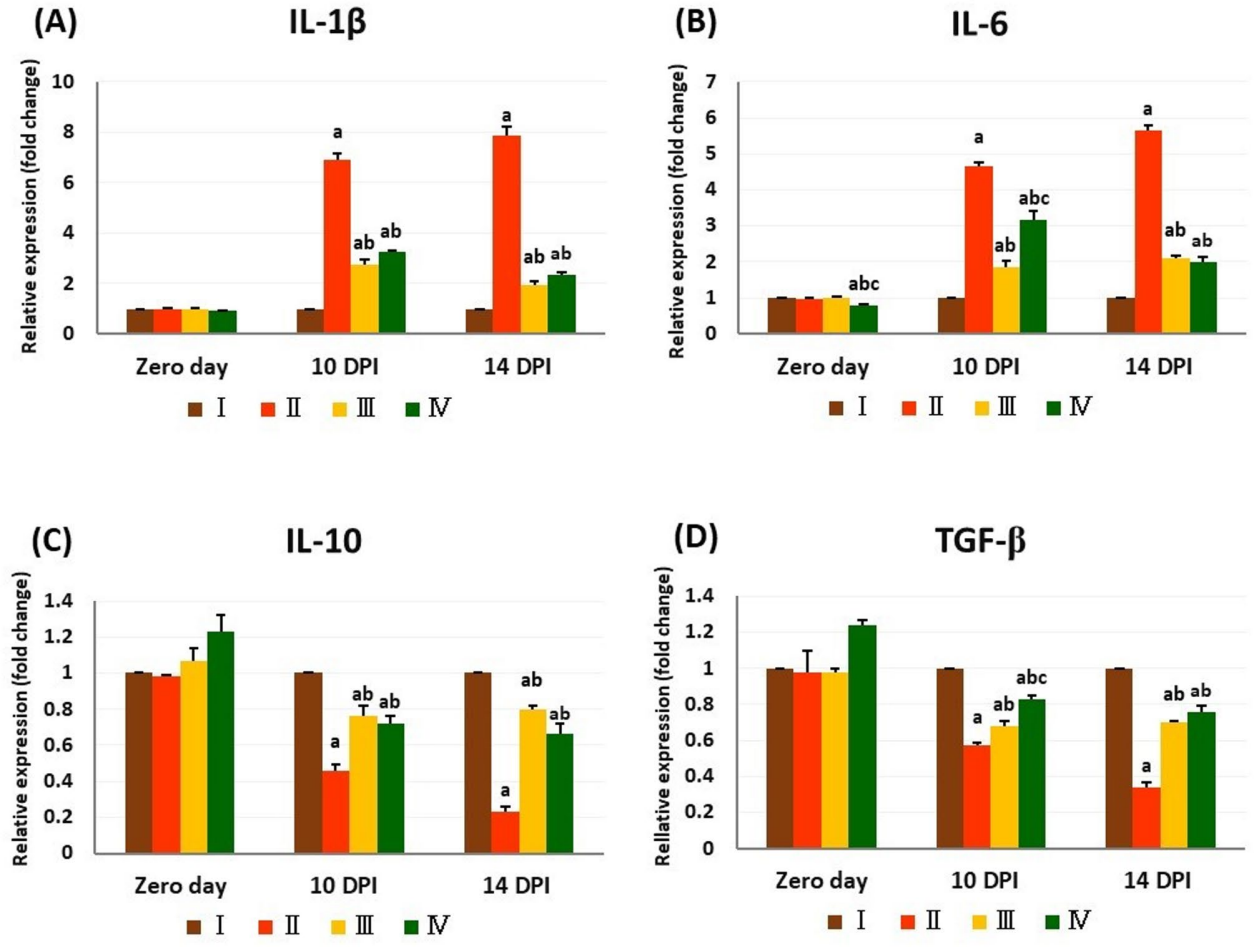
The relative expression levels of inflammatory cytokines in the blood of different experimental groups. The values are presented as means ± SE (*n* = 5) with different letters: (**a**) significant difference from group I; (**b**) significant difference from group II; (**c**) significant difference from group III; significant difference at *P < 0.05*. I: control negative group; II: control positive (infected) group; III: infected and treated with diminazene aceturate group; IV: treated with chicory fraction and infected group. IL-1β interleukin-1beta, IL-6 interleukin-6, IL-10 interleukin-10, TGF-β transforming growth factor beta, DPI day post-infection.

However, these responses showed marked improvement following the treatment of infected groups, as opposed to group II (infected and untreated). This was evidenced by significantly lower levels of IL-1β and IL-6 expression, alongside significantly higher levels of IL-10 and TGF-β expression in groups III and IV at the same time points (10^th^ and 14^th^ DPI), compared to group II. On the 10^th^ DPI, diminazene aceturate administration in group III caused a more noticeable decrease in expression levels of both TGF-β and IL-6 than chicory fraction administration in group IV (Fig. 4).

### Histopathological results

**Fig. 5:**
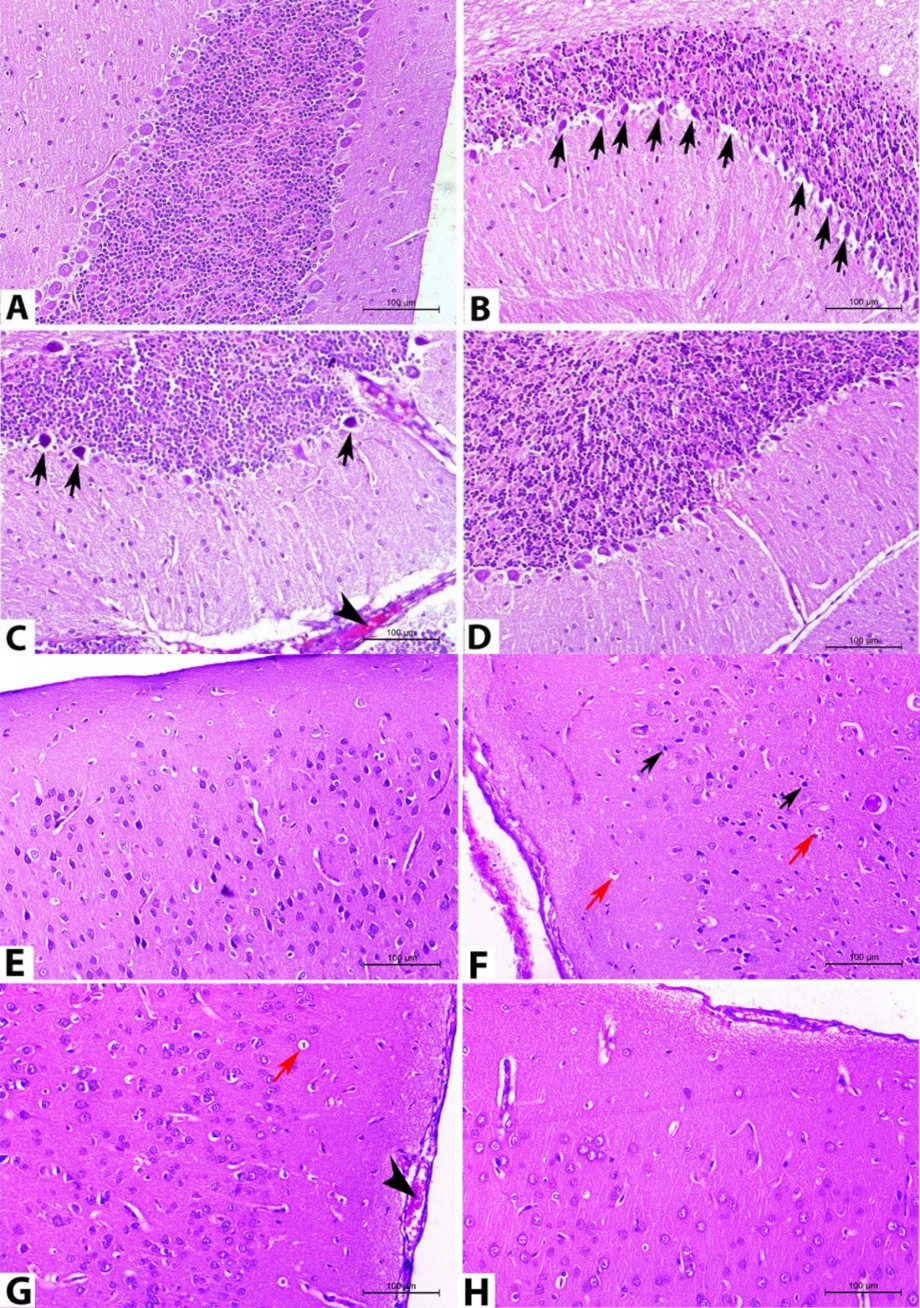
Photomicrographs of representative H&E-stained cerebellar and cerebral sections on the 14^th^ DPI. (**A**) the cerebellum of normal rats revealed a typical arrangement of the cerebellar cortex layer. (**B**) Cerebellar tissues of rats in group II showed multifocal necrosis and loss of Purkinje cell (arrow), granular cell layer atrophy. (**C**) Rats in group III showed similar cerebellar lesions but in a lesser degree of distribution and severity: necrosis of Purkinje cell (arrow) and congestion (arrowhead). (**D**) The cerebellum of rats in group IV revealed the above-described cerebellar neuropathology to group III but with more amelioration. (**E**) Normal rats showed the systematic arrangement of cerebral cortex layers. (**F**) The cerebrum in group II revealed disarrangement of cerebral cortical layers, pyknotic nuclei of some neurons (black arrow), and perivascular edema (red arrow). (**G**) Cerebral tissues of group III exhibited similar cerebral lesions, but in a lesser degree of severity: perivascular edema (red arrow) and congestion (arrowhead). (**H**) Rats in group IV had similar the above-described cerebral neuropathology to group III but with more improvement. (magnification 200X).

**Fig. 6:**
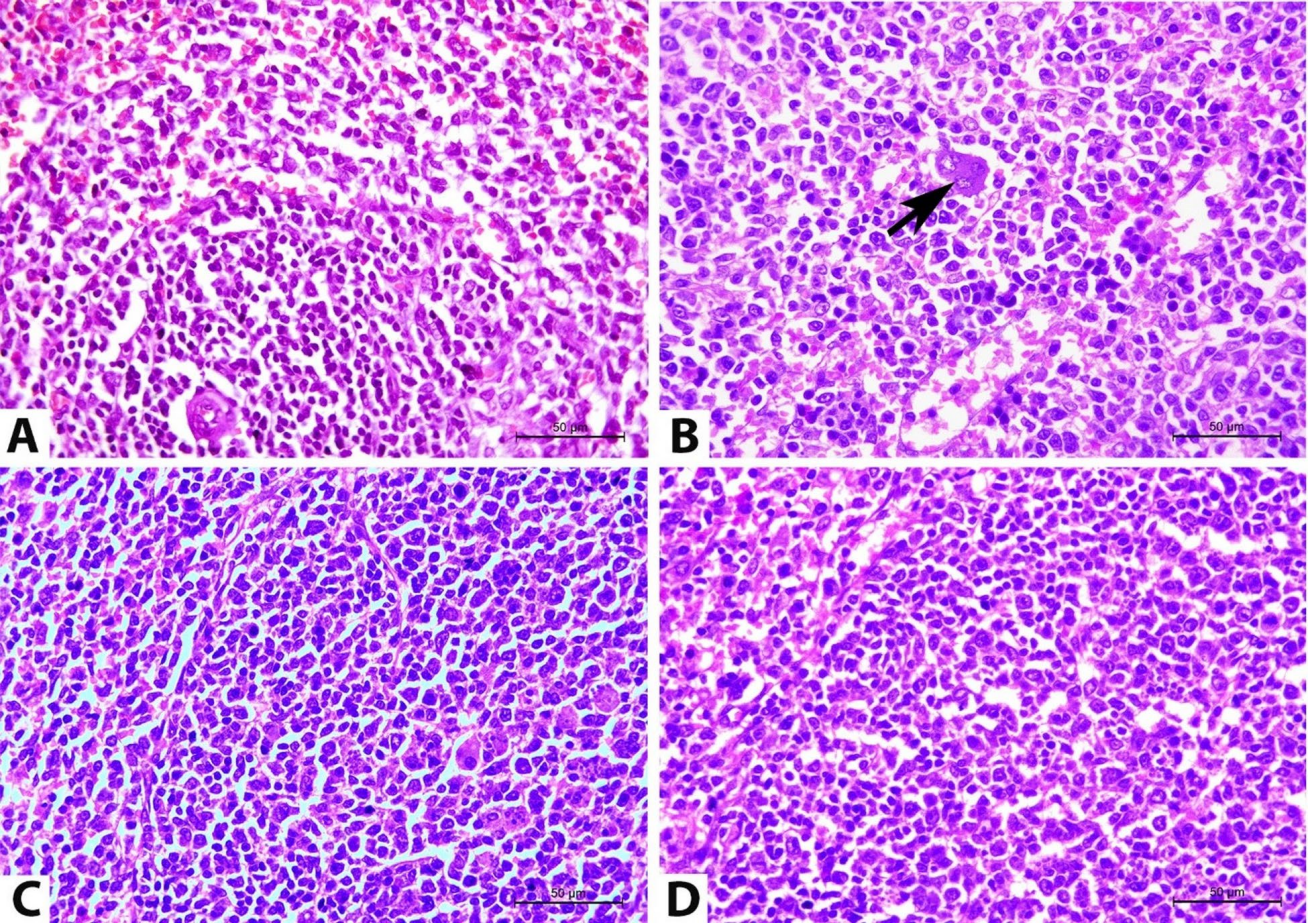
Photomicrographs of representative H&E-stained splenic sections on the 14^th^ DPI. (**A**) Rats in group I revealed the normal histological structure of the spleen; white and red pulps. (**B**) Rats in group II showed lymphoid and reticuloendothelial cell proliferation with several extra-medullary megakaryocytes (arrow). (**C**) Splenic tissue in groups III and (**D**) in IV exhibited improvement when compared to the rats in group II. (magnification 400X).

The alterations in the histopathology of cerebellum, cerebrum, and spleen on the 14^th^ DPI are presented in Figs. 5, 6.

In normal rats (group I), the cerebellar sections stained with hematoxylin and eosin (H&E) demonstrated the normal organized three layers of the cerebellar cortex, arranged from superficial to deep: the molecular layer, the Purkinje cell layer, and the granular cell layer (Fig. 5A). Conversely, in *T. evansi*-infected rats (group II), most of the cerebellar tissue sections showed Purkinje cell depletion and multifocal necrosis, along with congestion and noticeable granular cell layer atrophy, though the distribution pattern was not consistent across all fields that were examined (Fig. 5B). Conversely, the animals in group III displayed the previously mentioned cerebellar neuropathological alterations, albeit to a lesser degree and severity than those in group II (Fig. 5C). Rats in group IV exhibited similar neuropathology of the cerebellum but with significant improvement relative to those in groups II and III (Fig. 5D).

In normal rats (group I), cerebral sections exhibited the 6 layers of the cerebral cortex arranged in a systematic manner, these layers were identified from the outermost to the innermost as follows: the outer molecular layer, the outer granular layer, the outer pyramidal cell layer, the internal granular layer, the internal pyramidal layer, and the polymorphic cell layer (Fig. 5E). However, the cerebral cortical layers of *T. evansi*-infected rats (group II) were disrupted in the majority of their cerebral tissue sections There was cerebral blood vessel congestion, associated with neuronophagia, perivascular cuffing of mononuclear cells, perineuronal and perivascular edema, and gliosis. Numerous vacuolated and shrunken neurons were visible in the molecular layer. Several neurons exhibited karyolitic or pyknotic nuclei (Fig. 5F). Conversely, diminazene aceturate- treated rats (group III) showed the previously mentioned cerebral neuropathological lesions, the degree of severity and extent was lower in comparison to group II (Fig. 5G). Rats treated with chicory fraction (group IV) showed similar cerebral neuropathological changes to group III, but with greater alleviation (Fig. 5H).

The splenic sections of normal control rats (group I), stained with H&E, exhibited normal histology, including red and white pulps (Fig. 6A). In contrast, *T. evansi*- infected rats (group II) demonstrated an enlargement of splenic pulps attributable to an elevated proliferation of lymphoid and reticuloendothelial cells. Concurrently, certain rats exhibited necrosis of the white pulps and lymphoid depletion, associated with several apoptotic bodies. All spleen sections of both infected and uninfected rats contained extra-medullary megakaryocytes inside the red pulps; however, the number of these cells was noticeably higher in the infected rats than in the uninfected ones. In both the infected and uninfected rats, the hemosiderin pigment was not visible in the splenic tissue sections. The splenic blood vessels exhibited features of angiopathy, indicated by swelling and sloughing of the endothelium and vacuolar degeneration of tunica media (Fig. 6B). The spleen samples of rats in groups III (Fig. 6C) and IV (Fig. 6D) also showed these lesions, although they were less severe than group II. Rats in group III showed marked improvement, while group IV exhibited a moderate improvement compared to group II.

## Discussion

At the present time, *T. evansi* is considered a big challenge in countries with camel production, resulting in massive economic losses. The main problem with *T. evansi* infections is the resistance and toxicity related to current chemotherapies^42^. Therefore, natural products and their components hold a promise for novel drugs and alternative therapeutic approaches for the disease^22,43^.

In comparison to the untreated infected group, the administration of the chicory fraction could not eliminate trypanosomes from the blood, but it markedly decreased parasitemia degree. This reduction may be attributed to the presence of sesquiterpene lactones (49.9%), flavonoids (22.17%), phenolic acids (13.21%), and coumarin (7.11%) in this fraction. A recent report by Pena-Espinoza et al.^20^ proved the* in vitro* anti-trypanosomal efficacy of *C. intybus* against *T. cruzi* and elucidated the trypanocidal components, which include STLs (lactucin, dihydrolactucin, dihydro-8-deoxylactucin, 8-deoxylactucin dihydrolactucopicrin, lactucopicrin, and dihydrocostus lactone), flavonoids (luteolin, apigenin, and kaempferol), phenolic acids (caffeic acid, chicoric acid, chlorogenic acid, and esculetin), and fatty acids and their derivatives (13-docosenamide, 9-Oxo-ODE, 9(10)-EpOME, and 9-Octadecenamide). The trypanocidal modes of action of STLs potentially involve interaction with the protozoan’s sulfhydryl proteins and reduction of thioredoxin reductase (TR) activity leading to irreversible oxidation, as well as damage to proteins, DNA, and lipids, ultimately resulting in protozoan death through apoptosis, necrosis, and autophagy^44^.

On the other hand, the anti-trypanosomal efficacy of polyphenols (flavonoids and phenolic acids) may be due to macrophage activation, induction of morphological changes as chromatin condensation, DNA fragmentation, acidocalcisomes and glycosomes accumulation, Golgi apparatus damage, and mitochondrial dysfunction as well as dysregulation of mitochondrial enzymes and other enzymes essential for parasite survival such as arginase^45^.

The cardinal sign for *T. evansi* and another *Trypanosoma* spp.is anemia^46^. Hemolytic anemia concomitant with the infection may be the cause of the reduction in the erythrocytes, PCV, and Hb values in our study. Our findings agree with Darwish et al.^47^ and Razin et al.^48^. Potential reasons for red blood cell (RBC) destruction include the presence of the parasite and its metabolites in the blood, the hydrolyzing effect of trypanosomal sialidase enzyme on the membrane of red blood cells, escorted by the erythropoiesis inhibition, and oxidative damage of the RBCs^48,49^. Similar to Da Silva et al.^50^ and Do Carmo et al.^46^, the values of MCHC and MCV did not considerably vary across the experimental groups, indicating normocytic–normochromic anemia.

Leucocytosis, neutrophilia, lymphocytosis, and monocytosis related to the infection with *T. evansi*are compatible with the earlier study by Do Carmo et al.^46^. It may be an immunological reaction to the protozoan^47^. On the other hand, leucopenia linked to *T. evansi *infection in rats was reported by Razin et al.^48^.

The anti-inflammatory and trypanocidal properties may contribute to the modulation of inflammatory cytokines and hematological parameters, thereby reducing parasitemia and mitigating associated pathological changes.

Antibody-mediated phagocytosis, alongside pro-inflammatory cytokine production including IL-1, IL-6, and TNF-α, and nitric oxide secretion from classically activated macrophages, are the main mechanisms for removing the parasite from the bloodstream in trypanosomiasis^5^. Although inflammatory cytokines are pivotal in conferring disease resistance, their excessive production results in failure to control parasitemia and causes tissue damage^51^. IL-10 is necessary in influencing the outcome subsequent to the infection with *Trypanosoma*. Due to its anti-inflammatory properties, it regulates the hyperactivity of T-cells and macrophages, which are accountable for the pro-inflammatory cytokine production^5^. TGF-β is recognized as a versatile cytokine synthesized by different organs. It serves as an important immunosuppressive agent for the maintenance of immunological homeostasis. it exerts an inhibitory effect on the activity of immunocompetent cells, specifically impeding the differentiation of naïve T-cells into effector T-cells, namely CD8+ (cytotoxic) and CD4+ (helper) cells^52^.

The decrease in IL-6 and IL-1β, in addition to the elevation in TGF-β and IL-10 gene expression levels in rats treated with chicory fraction, underscores the anti-inflammatory properties of *C. intybus*, including STLs and their derivatives, as reported by previous studies^15,53,54^. The observed reduction in parasitemia in the chicory fraction-treated rats, could be attributed to the significant upregulation of IL-10 and TGF-β gene expression levels in addition to the antitrypanosomal efficacy of chicory components. According to Onyilagha and Uzonna^5^, the protection of *T. congolense*-infected cattle is related to elevated levels of IL-4 and IL-10 gene expression, as well as decreased nitric oxide. Furthermore, *T. brucei brucei*-infected mice that were deficient in CD8 + T cells exhibited a decreased parasitemia level in comparison to their wild-type counterparts.

According to the previous study conducted by Do Carmo et al.^46^, rats that were infected with *T. evansi* exhibited alterations in biochemical parameters, including reduction in blood glucose level, elevation in triglycerides and VLDL-C. These results are consistent with the current study’s findings. A frequent laboratory observation in trypanosomiasis is hypoglycemia, which results from excessive utilization of blood glucose by circulating trypanosomes and liver degeneration that impairs the gluconeogenic pathways in the liver^55^. The disease-related increase in VLDL-C and triglyceride levels may occur due to inhibition of the lipoprotein lipase enzyme through pro-inflammatory cytokines, which impairs the degradation of triglycerides^46,47^.

Our study’s findings on the reduction of HDL-C and LDL -C levels are consistent with those of Razin et al.^48^; nevertheless, Do Carmo et al.^46^ stated that the infection with *T. evansi* led to a statistically non-significant reduction in the HDL-C. These changes might be a result of parasite’s utilization of the host cholesterol and lipids, stemming from its incapacity to produce these components for its growth. Furthermore, this reduction in the HDL-C could be related to the oxidative stress coupled with the infection, as HDL-C possess antioxidant properties^47^.

The administration of *C. intybus* sesquiterpene lactones-enriched fraction and diminazene aceturate was observed to improve lipid profile and serum glucose levels. This enhancement is likely attributed to the reduction in the circulating trypanosomes in the blood, in addition to the antioxidant and anti-inflammatory properties of the compounds present in the chicory fraction.

Various studies examined the involvement of the central nervous system in trypanosomiasis pathophysiology^56,57^. Acetylcholine (ACh) undergoes hydrolysis by AChE enzyme, which controls the amount of this neurotransmitter within the synaptic cleft. As a result, it is important for mental processes, including memory and learning^58^. Our findings supported those of Baldissera et al.^59^ by exhibiting a significant increase in the activity of AChE related to the infection in groups II and III. *T. evansi* infection leads to a reduction in cerebral Na+, K+-ATPase activity, thereby inducing hyperpolarization of the neuronal cell membrane. This condition promotes the release of additional neurotransmitters, such as Ach. Therefore, the increase in AChE activity may be a compensatory response to the elevated levels of Ach neurotransmitter^60^. This change in neural activity may result from the parasite’s direct effects on the tissue of the brain or indirectly emerge as an inflammatory reaction to trypanosomes^59^, as confirmed, in the current study, by brain histopathological lesions.

The reduction of high AChE activity that approached the control level may be attributed to the acetylcholinesterase inhibitory properties of *C. intybus*^16^. On the other hand, the inability of diminazene aceturate to cross the blood-brain barrier might be the reason for the sustained increase in AChE activity observed in group III^61^.

Long-term exposure to the pathogen incites free radicals and the depletion of antioxidants, leading to oxidative stress and tissue damage^48^. The increase in MDA content coupled with the decrease in GSH concentration and GPx activity in the present study indicate that the *T. evansi*infection caused oxidative stress within brain tissue. These results are corroborated by a recent report from Dkhil et al.^57^, who demonstrated that the experimental infection of mice with *T. evansi* induced a marked elevation in the MDA levels with a concomitant decrease in the GSH concentrations within the cerebral tissue of the infected mice.

The administration of chicory fraction prominently improved the oxidative status of the brain, as various studies proved the antioxidant activities of *C. intybus*^53,62^. This antioxidant potential may contribute to the mitigation of RBC oxidative damage.

The pathological alterations in the spleen and brain, concurrent with *T. evansi* infection, have been shown by various reports^47,48,57^. These alterations are frequently due to the multiplication of the parasites within the bloodstream and their consumption of oxygen, which results in hypoxia and subsequent degenerative changes. Additionally, *T. evansi*’s released toxins and oxidative stress may play a role in these alterations^22,63^.

Megakaryocytes are a normal feature in the splenic tissue of rats as extra-medullary hematopoiesis (EMH)^64^. The marked increase of splenic extra-medullary megakaryocytes in the current study may be in response to *T. evansi*-induced anemia^65^. The angiopathy of blood vessels linked with the disease may be a contributing factor to this observation. Also, in the present study, the reticuloendothelial cell proliferation may be a compensatory mechanism to meet the need to destroy erythrocytes that were coated with the parasite antigens^63^. Trypanosome toxins may be the cause of the cellular apoptosis seen with infection. A recent report by Ramadan et al.^66^, stated that caspase-3 gene expression in spleens of *T. evansi*-infected mice was markedly upregulated, suggesting that it plays a role in apoptosis.

The chicory fraction administration alleviated the histopathological picture, potentially attributable to its anti-inflammatory, antioxidant, and anti-trypanosomal properties. The neuroprotective efficacy of *C. intybus* was proved by Al-Salim and Al-Charak^67^.

## Conclusion

Our results indicate that oral administration of a *C. intybus* fraction enriched with sesquiterpene lactones demonstrates significant *in vivo* anti-trypanosomal activity, as evidenced by reduced parasitemia levels and improvement of hematological, biochemical, oxidative, inflammatory, and histopathological alterations induced by *T. evansi*. Despite these promising findings, the present study represents a preliminary *in vivo* evaluation with certain limitations, including the relatively short experimental duration, and the absence of survival analysis. In addition, complete parasite clearance was not achieved during the study period. Therefore, the current results should be interpreted as foundational evidence rather than definitive therapeutic validation. Further studies are warranted to investigate long-term efficacy, survival outcomes, post-infection therapeutic protocols, dose optimization, pharmacokinetics, and combination therapy. It will also be of particular interest to evaluate the anti-trypanosomal activity of purified sesquiterpene lactones, as well as other bioactive constituents such as flavonoids and phenolic acids, which may act synergistically to enhance the overall therapeutic potential and confirm the clinical relevance of the observed anti-trypanosomal, anti-inflammatory, antioxidant, and protective effects.

## Supplementary Information

Below is the link to the electronic supplementary material.

Supplementary Material 1

## Data Availability

The data that support the findings of this study are available within the article.
