# Supplementary material for: Anti-trypanosomal, anti-inflammatory, and neuroprotective effects of Cichorium intybus sesquiterpene lactones in experimental Trypanosoma evansi infection
Source: Sci Rep. 2026 Apr 27;16:13522. doi: 10.1038/s41598-026-47119-z (PMC13121607; doi:10.1038/s41598-026-47119-z)
Supplement: Supplementary file 1 — Supplementary Material 1 [file 41598_2026_47119_MOESM1_ESM.docx]

**Anti-trypanosomal, anti-inflammatory, and neuroprotective effects of *Cichorium intybus* sesquiterpene lactones in experimental *Trypanosoma evansi* infection**

Marian G. Sawerus^*^, Hamdy H. Kamel, Walaa M. S. Ahmed, Sobhy Abdel-Shafy, Dalia El Amir, Emad A. Mahdi, Marwa A. Ibrahim, Olfat Shehata

^*^Corresponding author: Marian G. Sawerus

E-mails: [mariangamal95@yahoo.com](mailto:mariangamal95@yahoo.com)

[marian.gamal@vet.bsu.edu.eg](mailto:marian.gamal@vet.bsu.edu.eg)

**
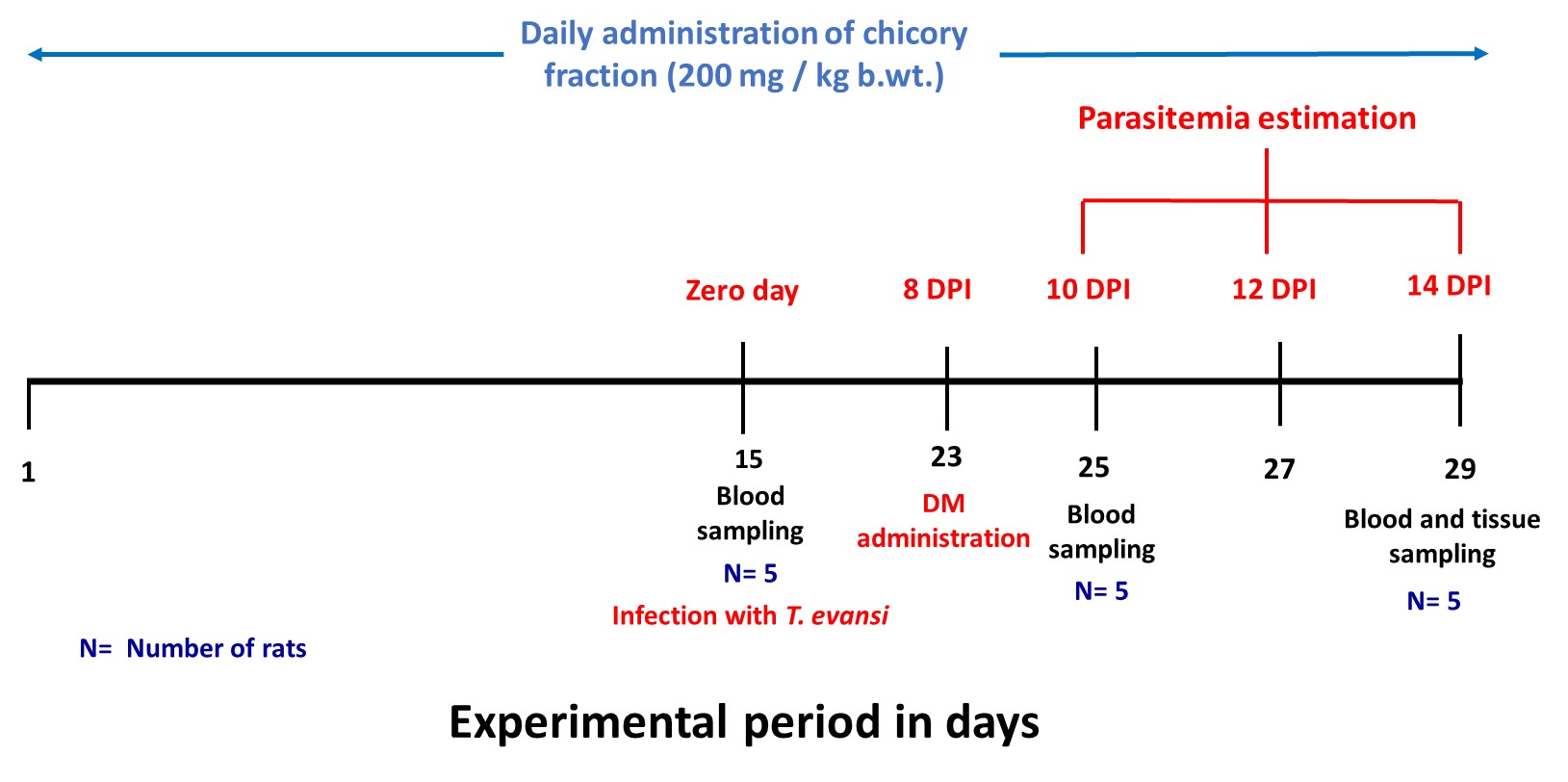
Supplementary Fig. 1: The experimental design and sampling.**

**Supplementary Table 1. Primers sequences used for quantitative real time PCR (qPCR).**

| Gene | Forward primer (5′ -3ˊ) | Reverse primer (5ˊ-3ˊ) | Amplicon size | References |
| --- | --- | --- | --- | --- |
| IL-1β | TTGAGTCTGCACAGTTCCCC | GTCCTGGGGAAGGCATTAGG | 161 | **^37^** |
| IL-6 | CCAGTTGCCTTCTTGGGACT | TCTGACAGTGCATCATCGCT | 224 | **^38^** |
| IL-10 | TCCCTGGGAGAGAAGCTGAA | CCTGCAGTCCAGTAGATGCC | 234 | **^39^** |
| TGF-β | CACTCCCGTGGCTTCTAGTG | GGACTGGCGAGCCTTAGTTT | 145 | **^40^** |
| Actb | CCGCGAGTACAACCTTCTTG | CAGTTGGTGACAATGCCGTG | 297 | **^41^** |

**Supplementary Table 2 Components of *C. intybus* fraction**

| Class | Components | Relative percentage (%) |
| --- | --- | --- |
| Sesquiterpene lactones | Dihydrolactucin | 14.11 |
|  | Lactucin | 13.26 |
|  | Dihydro-8-deoxylactucin (jaquinelin) | 7.17 |
|  | Lactucopicrin | 8.15 |
|  | Dihydrolactucopicrin | 4.04 |
|  | Dihydrocostus lactone | 3.17 |
| Flavonoids | Apigenin | 12.13 |
|  | Luteolin | 10.04 |
| Phenolic acids | Chicoric acid | 12.02 |
|  | Caffeic acid | 0.62 |
|  | Chlorogenic acid | 0.57 |
| Coumarin | Esculetin | 7.11 |
| Alkaloid | Scopolamine | 4.02 |
| Unidentified compounds |  | 3.59 |

Reported in Sawerus et al. **^21^**
